# XPO1 Inhibition Preferentially Disrupts the 3D Nuclear Organization of Telomeres in Tumor Cells

**DOI:** 10.1002/jcp.25378

**Published:** 2016-04-08

**Authors:** Cheryl Taylor‐Kashton, Daniel Lichtensztejn, Erkan Baloglu, William Senapedis, Sharon Shacham, Michael G. Kauffman, Rami Kotb, Sabine Mai

**Affiliations:** ^1^ Manitoba Institute of Cell BiologyCancerCare ManitobaUniversity of ManitobaWinnipegCanada; ^2^ Karyopharm Therapeutics Inc.NewtonMassachusetts; ^3^ CancerCare ManitobaWinnipegCanada

## Abstract

Previous work has shown that the three‐dimensional (3D) nuclear organization of telomeres is altered in cancer cells and the degree of alterations coincides with aggressiveness of disease. Nuclear pores are essential for spatial genome organization and gene regulation and XPO1 (exportin 1/CRM1) is the key nuclear export protein. The Selective Inhibitor of Nuclear Export (SINE) compounds developed by Karyopharm Therapeutics (KPT‐185, KPT‐330/selinexor, and KPT‐8602) inhibit XPO1 nuclear export function. In this study, we investigated whether XPO1 inhibition has downstream effects on the 3D nuclear organization of the genome. This was assessed by measuring the 3D telomeric architecture of normal and tumor cells in vitro and ex vivo. Our data demonstrate for the first time a rapid and preferential disruption of the 3D nuclear organization of telomeres in tumor cell lines and in primary cells ex vivo derived from treatment‐naïve newly diagnosed multiple myeloma patients. Normal primary cells in culture as well as healthy lymphocyte control cells from the same patients were minimally affected. Using both lymphoid and non‐lymphoid tumor cell lines, we found that the downstream effects on the 3D nuclear telomere structure are independent of tumor type. We conclude that the 3D nuclear organization of telomeres is a sensitive indicator of cellular response when treated with XPO1 inhibitors. J. Cell. Physiol. 231: 2711–2719, 2016. © 2016 The Authors. *Journal of Cellular Physiology* published by Wiley Periodicals, Inc.

Telomeres are at the ends of chromosomes and key to chromosomal stability (for review, see Mai, 2010). A protein complex termed shelterin caps intact telomeres and prevents genomic instability by protecting telomeric ends from DNA damage signaling, illegitimate recombination and fusions. Disruption of shelterin is found in cancer cells and leads to a dynamic process of ongoing instability and generates heterogeneous tumor cell populations (Mai, 2010; Lajoie et al., 2015).

In the past decade, our group has demonstrated that telomeres display a defined order in normal cells and undergo dynamic changes in cancer cells (Chuang et al., 2004; Knecht et al., 2009; Gadji et al., 2010, 2012; Knecht et al., 2012; Samassekou et al., 2013). These changes are quantitated using TeloView, a program we developed to specifically assess the 3D telomeric profile of each nucleus (Vermolen et al., 2005). Using TeloView, we measured significant 3D nuclear telomere alterations in multiple tumor types, including glioblastoma, prostate cancer, Hodgkin's lymphoma, myelodysplastic syndromes, acute and chronic myeloid leukemias. These 3D telomeric profiles were indicative of stable or progressive disease.

Exportin‐1 (XPO1), also known as chromosome region maintenance 1 protein (CRM1), is a key nuclear‐cytoplasmic transport protein that exports a broad range of cargo proteins from the nucleus to the cytoplasm of a cell (Fornerod et al., 1997; Fukuda et al., 1997; Nguyen et al., 2012). XPO1 is involved with the export of more than 200 nuclear proteins including p53, IκB, and FOXO3a (Xu et al., 2012). In addition several tumors types have been shown to have increased expression of XPO1 when compared to their normal cell counterparts (Senapedis et al., 2014). Karyopharm Therapeutics has developed a series of small‐molecule Selective Inhibitor of Nuclear Export (SINE) compounds that block XPO1 function both in vitro and in vivo (Senapedis et al., 2014). The clinical compound selinexor (KPT‐330), is currently in Phase‐II/IIb clinical trials for treatment of both hematologic and solid tumors. As of March 2016 over 1400 patients have been treated with selinexor. KPT‐8602 is the second generation XPO1 inhibitor and is in human clinical trials for the treatment of multiple myeloma.

This study examines whether XPO1 inhibition can affect the 3D nuclear telomere organization. To study this question, we used tumor cell lines of lymphoid origin (Raji and Jurkat) and of epithelial origin (breast cancer cell lines T47D and HCC1937) as well as primary human fibroblasts (BJ5ta). To validate the cell line findings, we investigated myeloma cells of treatment‐naïve patients at diagnosis and their healthy control lymphocytes ex vivo. In this study we found that XPO1 inhibition preferentially affects tumor cells by disrupting their 3D nuclear telomere organization, while normal cells are minimally affected.

## Materials and Methods

### Cell lines and cell culture

The T cell lymphoma line Jurkat, the Burkitt's lymphoma line Raji, and the breast cancer cell lines T47D and HCC1937 were cultivated in RPMI1640 (Life Technologies, Burlington, ON, Canada) supplemented with 1% Na pyruvate, 1% L‐glutamine, 1% Penicillin/streptomycin, 10% Fetal Bovine Serum at 5%CO_2_ in a humidified incubator at 37°C. Primary human fibroblasts (Bj5ta, ATTC, http://www.atcc.org/) were grown at 5%CO_2_ in a humidified incubator at 37°C as described by the supplier using a 4:1 mixture of Dulbecco's medium and Medium 199 (Life Technologies, Burlington, ON, Canada) with supplements as follows: 4 parts of Dulbecco's Modified Eagle's Medium containing 4 mM L‐glutamine, 4.5 g/L glucose, and 1.5 g/L sodium bicarbonate 1 part of Medium 199 supplemented with: 0.01 mg/ml hygromycin B and 10% fetal bovine serum.

### Ex vivo study of myeloma cells and control lymphocytes

#### Ethics

Ethics approval (HS10953 [H2010:170]) was obtained for the study and informed consent obtained from all patients. Patient characteristics are summarized in Table 1. All patients were treatment naïve.

**Table 1:** Clinical information of the 10 treatment‐naïve patients who donated samples for this study

Patient number	Sample date	Age at diagnosis	Sex	BMPCS %	Date BMPCS%	IgG	IgA	IgM	Light chain isotype (kappa/lambda)	Kappa SFLC	Lambda SFLC	Ratio SFLC	M band g/L
1	6/2/15	68	M	16.2	11/29/11	20.80	0.80	0.62	IgG kappa	487.50	11.20	43.53	14 g/L
2	6/29/15	75	F	9.6	8/29/11	22.20	1.04	0.77	IgG kappa	No light chains noted	No light chains noted	No light chains noted	Two M bands 3.7 g/L and 8.0 g/L noted, both IgG kappa
3	7/7/15	70	F	26.8	2/20/13	32.22	1.08	1.42	IgG kappa	890.00	9.09	97.91	29 g/L
4	5/25/15	61	M	11.4	3/21/14	27.60	3.13	0.35	IgG kappa	29.30	21.10	1.39	16 g/L
5	8/12/15	73	M	16.4	4/30/13	4.42	11.60	0.30	IgA lambda	Testing not done	Testing not done	Not done 13 g/L	
6	8/24/15	70	M	37.4	7/19/11	2.32	32.60	0.06	IgA kappa	Not present	Not present	Not present	19 g/L
7	9/8/15	66	F	29.8	8/20/15	4.28	16.60	0.19	IgA kappa	21.58	1.63	13.24	12 g/L
8	9/16/15	65	F	1.6	6/3/15	18.00	1.74	0.76	IgG kappa	143.04	4.43	32.29	10 g/L
9	9/22/15	76	M	68.0	7/31/15	2.96	0.08	<0.04	IgD lambda	Testing not done	Testing not done	Not done 11 g/L	
10	6/3/15	56	F	8.2	4/15/13	29.50	0.25	0.46	IgG lambda	<2.70	11.70	0.23	25 g/L

Diagnosis: 1, IgG kappa smouldering multiple myeloma; 2, smouldering multiple myeloma; 3, smouldering multiple myeloma; 4, smouldering multiple myeloma; 5, smouldering multiple myeloma; 6, IgA kappa smouldering multiple myeloma; 7, multiple myeloma; 8, multiple myeloma; 9, ISS‐I multiple myeloma; 10, smouldering multiple myeloma.

### Isolation of lymphocytes and myeloma cells from patient blood

Ten milliliters of patient blood was kept at 37°C (up to 21 h) and then control lymphocytes and myeloma cells were isolated using Ficoll‐Paque (GE Healthcare, Piscataway, NJ) separation. Blood was overlaid on 5 ml of Ficoll‐Paque then centrifuged at 200*g* for 30 min at room temperature with acceleration and brake set to 3 on Eppendorf Model 5810R table top centrifuge (Fisher Scientific, Burlington, ON, Canada). Buffy coat was extracted, mixed with 14 ml of 1× phosphate buffered saline (PBS) and then centrifuged at 1200 rpm (290*g*) for 10 min. If red blood cells remained, the sample was treated with ACK buffer (150 mM NH_4_CL/10 mM KHCO_3_/0.1 mM EDTA.Na_2_‐2H_2_O) to lyse RBCs and centrifuged at 1200 rpm (290*g*) for 5 min at room temperature. Cell pellet was reconstituted in 2 ml of 1× PBS and cells were counted on a haemocytometer.

### Cell culture of primary lymphocytes and myeloma cells

No less than one million cells were plated in 5 × 10 cm cell culture dishes with RPMI 1640 media (supplemented with 1% Na pyruvate, 1% L‐glutamine, 1% Penicillin/streptomycin, 10% Fetal Bovine Serum).

### Treatment scheme of cell lines and primary cells with SINE compounds

One micromolar of each of the SINE (KPT‐185, KPT‐330/selinexor, KPT‐8602) compounds or DMSO or nothing was added to each cell culture plate: (a) cells + KPT‐330; (b) cells + KPT‐185; (c) cells + KPT‐8602; (d) cells + DMSO; and (e) cells alone. Cells plus compounds or controls were incubated at 37°C, 5% CO_2_ for 4 h.

### Cell harvest and fixation

Cells from each plate were harvested and centrifuged for 5 min at 1200 rpm (290*g*), then washed one time with 1× PBS and centrifuged for 5 min at 1200 rpm (290*g*). Cells were reconstituted in 1× PBS and then 100,000 cells were pipetted onto glass microscope slides and briefly air dried for 5 min. Slide samples were fixed for 10 min in 3.7% formaldehyde/1× PBS, then washed three times with 1× PBS on a shaking platform. Samples were then dehydrated for 3 min each in 70%, 90%, and 100% ethanol. Slides were air dried for 5 min and then stored at −20°C until immuno‐FISH was performed.

### 3D immuno‐staining and quantitative fluorescent in situ hybridization (3D‐qFISH)

Cell sample slides rehydrated and were fixed in 3.7% formaldehyde/1 × PBS for 10 min at room temperature then washed twice in 1× PBS for 5 min each at room temperature on a shaking platform. Slides were blocked with 4%BSA/4 × SSC for 5 min and then mouse anti‐human CD138 antibody conjugated to FITC (BD Biosciences, Mississauga, ON, Canada) was diluted 1:20 in 4%BSA/4 × SSC and incubated with slides for 60 min in a humid chamber at 37°C. Slides were washed three times in 1× PBS for 5 min each at room temperature on a shaking platform. Six microliters of PNA‐telomere probe (DAKO Diagnostics, Mississauga, ON, Canada) was added to slides under a 24 × 24 mm glass cover slip and sealed with rubber cement. Hybridization was performed on a Hybrite™ (Vysis/Abbott); denaturation was performed for 3 min at 80°C, hybridization took place for 2 h at 30°C. Post‐hybridization, slides were washed in 70% formamide/10 mM Tris (pH 7.4) twice for 15 min at room temperature on a shaking platform, then one minute in 1× PBS, 5 min in 0.1× SSC at 55°C and twice for 5 min each in 2× SSC/0.05% Tween 20 at room temperature on a shaking platform. The primary antibody (mouse anti‐human CD138‐FITC, 1:20 dilution in 4%BSA/4 × SSC) was re‐applied and incubated on slides an additional 60 min in a humid chamber at 37°C. Antibody was removed with three 5 min‐washes at room temperature in 1× PBS and then slides were stained with DAPI (0.1 μg/ml) for 3 min. Slides were mounted in Vectashield (Vector Laboratories Inc., Burlington, ON, Canada) and imaged.

### 3D imaging

30 cells from each sample were imaged on a Zeiss AxioImager Z2 (Carl Zeiss Canada, ON, Canada) with the AxioVision 4.8 software, taking 40 200 μm z‐stacks. An AxioCam HR charge‐coupled device (Carl Zeiss) with a 63×/1.4 oil objective lens (Carl Zeiss) and DAPI and Cy3 filters for detection of nuclear DNA staining and telomere probe signals, respectively (Carl Zeiss). Image acquisition for Cy3 was kept constant throughout the analysis to ensure comparison between experimental arms. 3D images were generated using the iterative deconvolution algorithm (Schaefer et al., 2001).

### 3D quantitative image analysis

3D images were analyzed using the TeloView™ program (Vermolen et al., 2005). TeloView is proprietary to 3D Signatures Inc. and was used with the company's permission. This program measures the (in) stability of the genome by assessing the numbers and intensity of each telomeric signal present per nucleus, which are indicative of the ploidy of the nucleus and the length of each telomere, respectively. In addition, the number of telomeric aggregates is determined. These are clusters of telomeres found in tumor cells (Mai and Garini, 2006) that at an optical resolution limit of 200 nm cannot be separated as individual signals. Furthermore, the cell cycle stage is measured using the *a/c* ratio that is indicative of resting or dividing cells (Table 2 and Vermolen et al., 2005; Gadji et al., 2010). Finally, TeloView determines the nuclear volume, and the number of telomeres per nuclear volume.

**Table 2:** Overview of 3D telomere parameters measured by TeloView

Measured feature	Read‐out	References
Number of signals	Number of telomeres indicative of genome stability or degree of genomic instability	Vermolen et al., (2005), Chuang et al. (2004), De Vos et al. (2009), Mai (2010), Gadji et al. (2010), Gadji et al. (2012), Knecht et al. (2012), Knecht et al. (2013)
Number of aggregates	Number of telomeric clusters found in a nucleus at 200 nm optical resolution	Vermolen et al. (2005), Louis et al. (2005), Mai and Garini (2006)
Signal intensity	Relative fluorescent intensity of telomeric signals indicative of telomere length	Poon et al. (1999)
*a/c* ratio	Overall nuclear distribution of telomeres, also measures a cell cycle dependent profile	Vermolen et al. (2005), Gadji et al. (2010)
Nuclear volume	3D measurement of nuclear size	Vermolen et al. (2005)

### Statistical analysis

Statistical analysis included frequency procedures for signal intensities using Chi‐Square, Likelihood ratio Chi‐Square and Mantel‐Haenszel Chi Square analyses. Group summary variables were compared using the GLM procedure and least squares means for all other parameters measured with TeloView (Vermolen et al., 2005). *P* < 0.05 was considered significant.

## Results

### In vitro analysis of lymphoid tumor cell lines treated with SINE compounds

**Figure 1:**
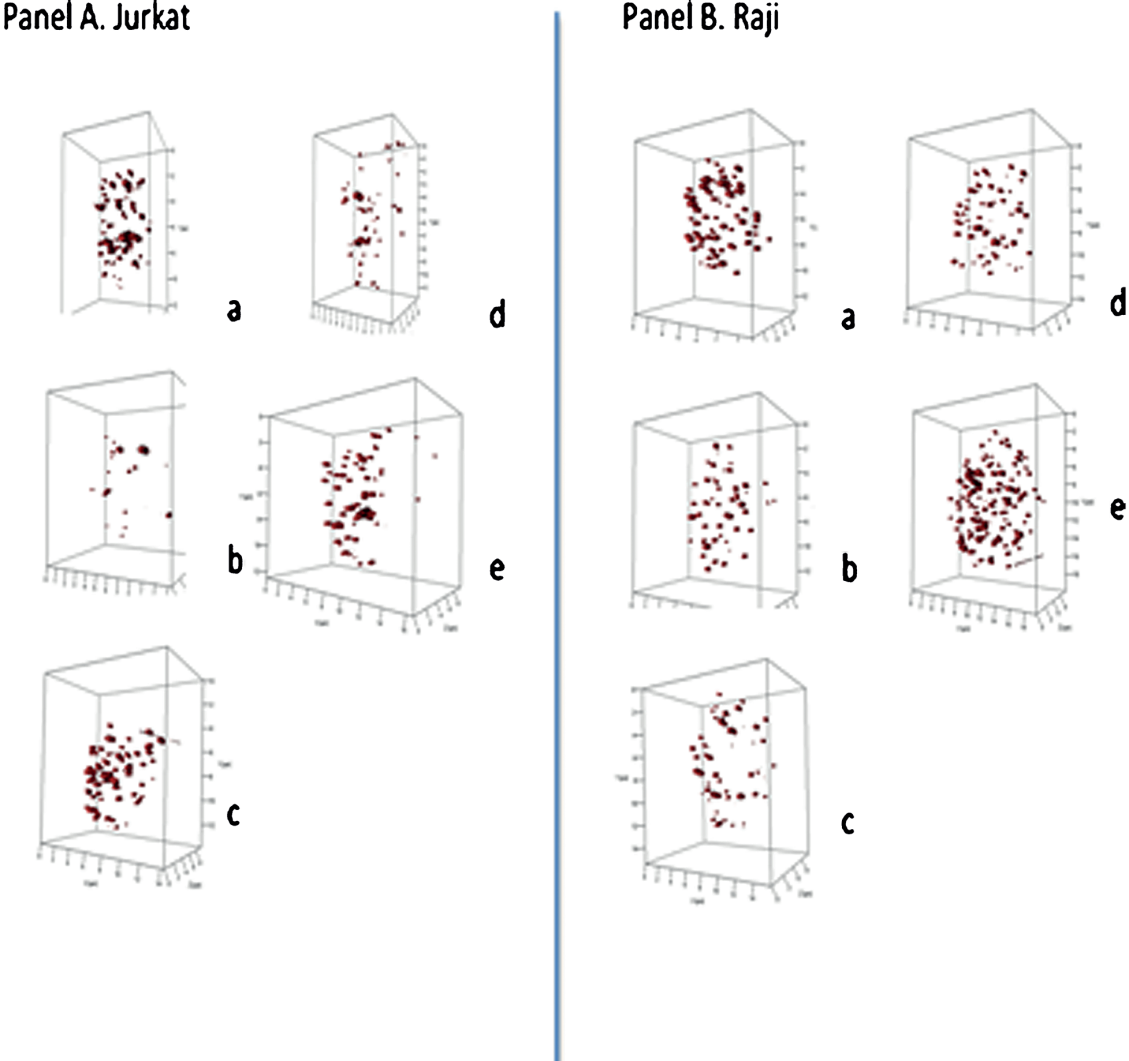
Three‐dimensional (3D) profiles of telomere organization in representative nuclei from Jurkat (Panel A) and Raji (Panel B). 3D profiles show telomeric signals (red) in selected nuclei for control cells (Panels A, B, **a**), after DMSO (Panels A, B, **b**), following KPT‐185 (Panels A, B, **c**), KPT‐330 (Panels A.B, **d**), and KPT‐8602 (Panels A,B, **e**).

We first analyzed pairs of cell lines of lymphoid origin after treatment with SINE compounds. The lymphoid cell line Jurkat (T cell lymphoma) and Raji (Burkitt's lymphoma) were treated with KPT‐185, KPT‐330, and KPT‐8602 (Materials and methods) and the telomere structure was examined. Figure 1 gives a representative overview of the 3D nuclear telomeric images in two‐dimensional (2D) and 3D display for Jurkat (Figure 1, Panel A) and Raji (Figure 1, Panel B) before and after treatment. All SINE compounds (but not DMSO) induce significant changes in the organization of 3D nuclear telomeres throughout the 4 h‐treatment period as measured by our quantitative software TeloView (Vermolen et al., 2005). Table 2 summarizes the 3D telomeric features that were measured in this study. The following changes in the 3D nuclear telomere organization were observed as a result of SINE compound treatment. In both Raji and Jurkat cells, the number of telomeric signals and the total number of telomeric aggregates were changed significantly (*P* < 0.001 for Jurkat; *P* < 0.05 in Raji). Additionally, the nuclear distribution of telomeres measured by the *a/c* ratio, which is indicative of cell cycle distribution (Vermolen et al., 2005, and Table 2) changed significantly for Jurkat only (*P* < 0.001). The nuclear distribution of the average number of signals was significantly altered in Jurkat only (*P* < 0.05), while the distribution of total intensity changed in both cell lines (Jurkat and Raji, both *P* < 0.001). Finally, nuclear volume as well as telomeres per nuclear volume were significantly affected by SINE compounds (*P* < 0.001 for both).

Not all SINE compounds showed identical effects on these 3D nuclear telomere parameters. For example, in Jurkat cells, KPT‐185 was more efficient in the induction of an altered distribution of the total number of telomeric signals and in the induction of aggregates than KPT‐8602, while KPT‐8602 significantly changed the *a/c* ratio as compared to KPT‐185. Overall, the above data indicate that the 3D nuclear telomere organization of both cell lines was affected by the SINE compounds. However, Jurkat cells were more sensitive to these compounds than Raji cells.

### In vitro analysis of breast cancer cell lines treated with SINE compounds

**Figure 2:**
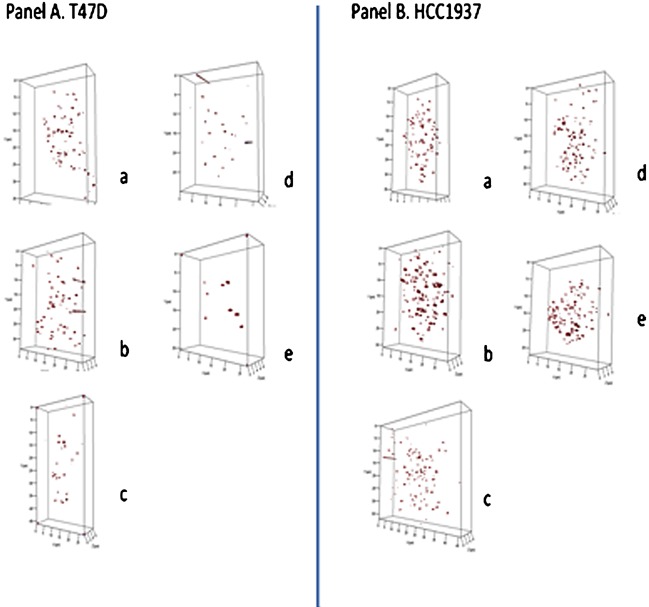
Three‐dimensional (3D) profiles of telomere organization in representative nuclei from T47D (Panel A) and HCC1937 (Panel B). 3D profiles show telomeric signals (red) in selected nuclei for control cells (Panels A, B, **a**), after DMSO (Panels A, B, **b**), following KPT‐185 (Panels A,B, **c**), KPT‐330 (Panels A.B, **d**), and KPT‐8602 (Panels A,B, **e**).

To determine if these changes were universal to cancer and not restricted to lymphoid cells, we analyzed the breast cancer cell lines T47D and HCC1937 (with BRCA1 mutation). Both cell lines reacted with 3D telomere alterations as a result of the 4 hour‐SINE compound treatments (Fig. 2, Panels A and B, respectively). While both cell lines were affected by XPO1 inhibition, T47D cells were less sensitive than HCC1937.

The total number of telomeric signals detected was significantly altered for both cell lines (*P* < 0.009). The distribution of telomeric aggregates was changed in HCC1937 (*P* < 0.0001), while no significant changes of this parameter were noted in T47D. The average number of telomeric signals increased significantly for both as did the total intensity and the nuclear volume measured (*P* < 0.001).

### In vitro analysis of primary human fibroblasts treated with SINE compounds

**Figure 3:**
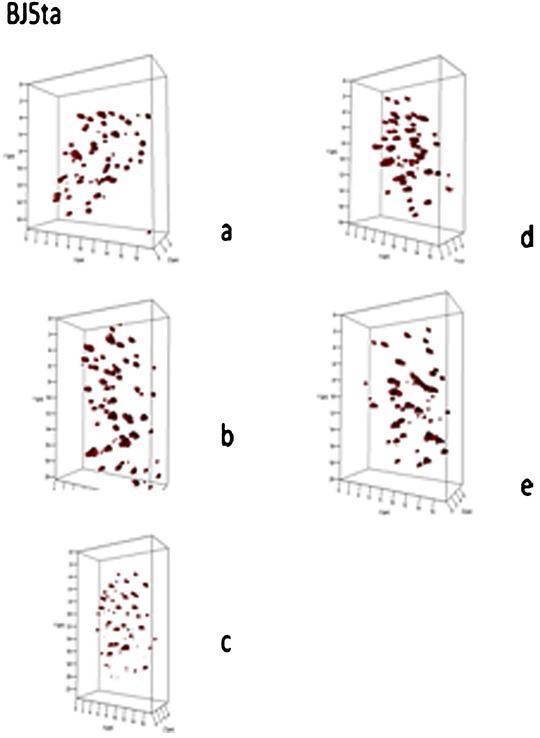
Three‐dimensional (3D) profiles of telomere organization in representative nuclei from BJ5ta. 3D profiles show telomeric signals (red) in selected nuclei for control cells (**a**), after DMSO (**b**), following KPT‐185 (**c**), KPT‐330 (**d**), and KPT‐8602 (**e**).

We next investigated whether or not primary human fibroblast cells (Bj5ta) displayed similar effects when treated with SINE compounds (Fig. 3). To our surprise, Bj5ta cells were insensitive to SINE treatment. The total number of signals and aggregates detected remained the same. It is of note that the *a/c* ratio that shows cell cycle distribution was changed suggesting cell cycle arrest as a result of treatment (*P* < 0.0001). Similarly, nuclear volume and telomeres per nuclear volume were changed in conjunction with the cell cycle arrest. These data suggest that normal primary fibroblasts do not respond in the same way tumor cells do to the disruption of their 3D nuclear telomere architecture when treated with up to 1 μM KPT‐185, KPT‐330 or KPT‐8602 over a 4 h‐period.

### Ex vivo study of human myeloma cells

The in vitro study of XPO1 inhibition suggested a preference of XPO1‐inhibition‐mediated 3D nuclear telomere disruption in tumor cell lines. In order to confirm that these observations were not cell culture artifacts, we decided to perform an ex vivo study with 10 treatment‐naïve myeloma patients who had consented to the study at diagnosis. Patient characteristics are summarized in Table 1.

After the isolation of myeloma cells and lymphocytes from the patients’ blood, the cells were cultivated as described in Materials and Methods and exposed to KPT‐185, KPT‐330, or KPT‐8602. At baseline, normal lymphocytes and CD138+ myeloma cells displayed significant differences in their 3D nuclear telomere organization (*P* < 0.001). These findings were expected as tumor cells and normal cells differ in their 3D nuclear architecture (Mai, 2010; Gadji et al., 2011).

After treatment, 3D fixation and quantitative imaging analysis of CD138+ myeloma cells and healthy control lymphocytes, we found that all patients showed a response to one, two or three SINE compounds for their myeloma cells (Table 2). In some patients, we noted a differential response with respect to SINE compounds used to disrupt the 3D nuclear architecture. In contrast, the healthy control lymphocytes were not or very moderately affected (*P* > 0.05 for all telomere parameters, except average intensity of all signals (*P* = 0.02 in patient 8), telomeres/nuclear volume (*P* = 0.007 in patient 9), average intensity and telomeres/nuclear volume (*P* < 0.05 in patients 1, 6, and 10) concomitant with total telomere intensity in patients 4 and 5.

**Figure 4:**
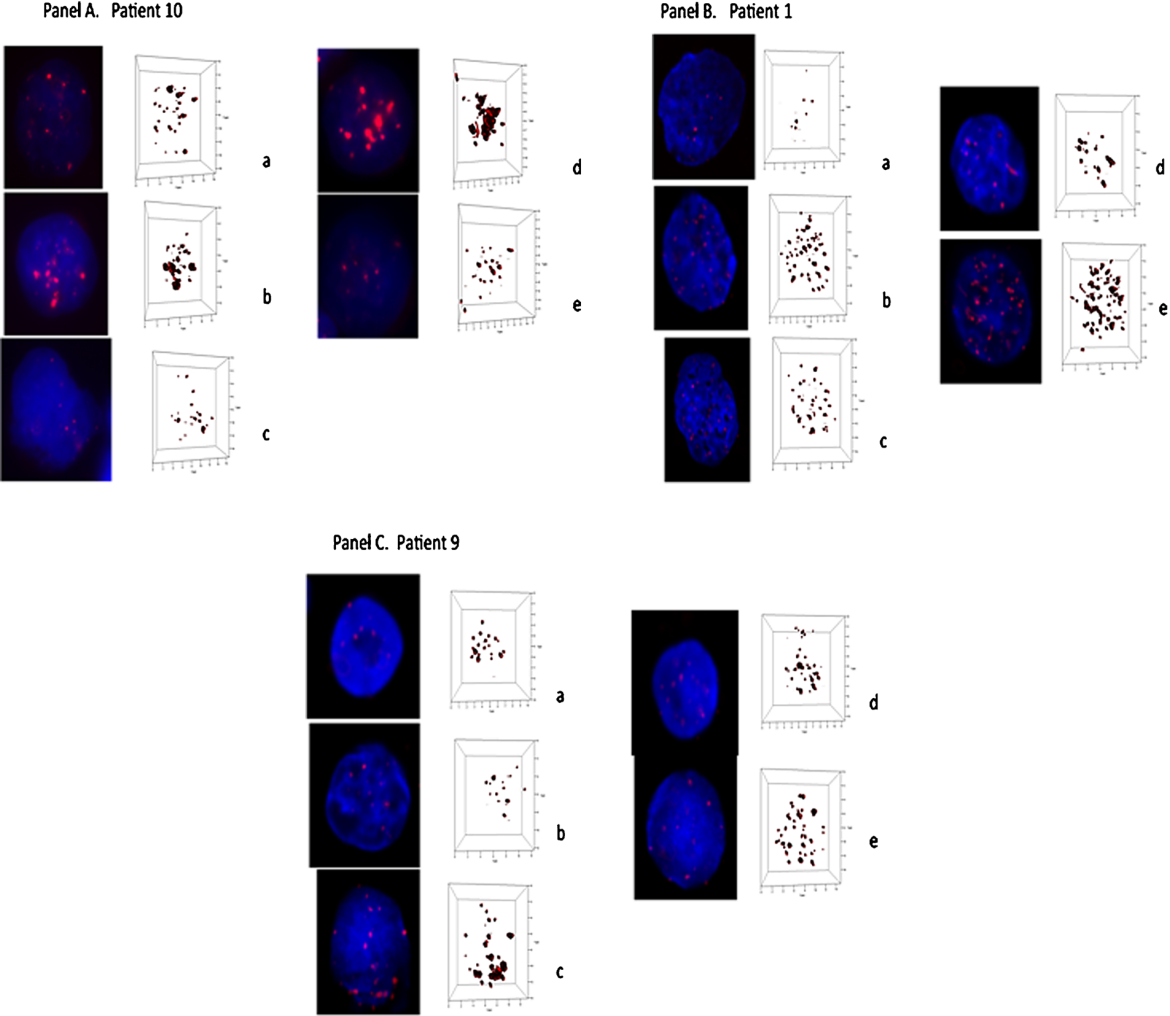
Three‐dimensional (3D) profiles of telomere organization in representative nuclei from treatment‐naïve myeloma patients; patient10 (Panel A), patient 1 (Panel B), and patient 9 (Panel C).

Figure 4 (panel A) gives an overview of preferential 3D telomere disruption by KPT‐330 in patient 10 (Table 1), while Figure 4 (panels B and C) show examples of patients who preferentially respond to KPT‐8602 (patient 1) or KPT‐185 (patient 9), respectively. Note that some patients respond equally well to two or all compounds as summarized in Table 3.

**Table 3:** Summary of response of CD138+ myeloma cells ex vivo to KPT‐185, KPT‐330, or KPT‐8602

	SINE compound			
Parameter measured	KPT‐330	KPT‐185	KPT‐8602	Patient number
Total number of signals	I 0.027	I 0.039	I <0.0001	1
	I <0.0001	ns	ns	10
	ns	I 0.014	I <0.0001	4
	I 0.016	D <0.0001	ns	2
	I 0.022	ns	ns	3
	ns	D 0.043	ns	5
	ns	n.d.	ns	6
	ns	n.d.	n.d.	8
	I 0.0008	ns	ns	7
	ns	I <0.0001	ns	9
Total number of aggregates	I 0.006	ns	I 0.0034	1
	I 0.008	ns	ns	10
	ns	ns	I 0.0123	4
	ns	D <0.0001	D 0.0014	2
	ns	ns	ns	3
	ns	ns	ns	5
	ns	n.d.	D 0.042	6
	ns	n.d.	n.d.	8
	I 0.036	ns	ns	7
	ns	I 0.005	ns	9
*a/c* ratio	ns	ns	I 0.006	1
	I 0.0005	1 0.0002	I 0.047	10
	D 0.003	D 0.0005	ns	4
	ns	D 0.006	ns	2
	ns	ns	ns	3
	I 0.003	D .012	D 0.004	5
	D 0.0002	n.d.	D <0.0001	6
	I 0.016	n.d.	n.d.	8
	ns	ns	ns	7
	ns	I 0.0029	ns	9
Average intensity	I <0.0001	I 0.0003	I 0.016	1
	ns	I <0.0001	I <0.0001	10
	ns	D 0.012	ns	4
	ns	D <0.0001	ns	2
	I <0.0001	ns	ns	3
	ns	ns	ns	5
	ns	n.d.	I 0.001	6
	D 0.0003	n.d.	n.d.	8
	D 0.018	ns	ns	7
	D 0.035	ns	ns	9
Total intensity	I <0.0001	I <0.0001	I <0.0001	1
	I 0.004	D <0.0001	D 0.0022	10
	ns	ns	I 0.0004	4
	ns	D <0.0001	ns	2
	I <0.0001	ns	ns	3
	ns	ns	ns	5
	ns	n.d.	I 0.005	6
	D 0.0013	n.d.	n.d.	8
	I 0.045	I 0.017	I 0.0023	7
	ns	I <0.0001	ns	9
Nuclear volume	ns	ns	I <0.0001	1
	ns	ns	ns	10
	D 0.0065	ns	ns	4
	ns	ns	ns	2
	ns	ns	ns	3
	ns	ns	I 0.0051	5
	ns	n.d.	ns	6
	I .004	n.d.	n.d.	8
	I <0.0001	D <0.0001	ns	7
	I 0.0013	I <0.0001	ns	9
Telomeres/nuclear volume	ns	I 0.014	ns	1
	I 0.0007	ns	ns	10
	I 0.019	ns	I .003	4
	ns	D <0.0001	D 0.011	2
	I 0.0002	ns	ns	3
	ns	ns	ns	5
	ns	n.d.	ns	6
	ns	n.d.	n.d.	8
	ns	I <0.0001	I 0.022	7
	ns	ns	ns	9
Intensity ≤7000	ns	ns	D 9%	1
	ns	ns	ns	10
	ns	ns	ns	4
	ns	I 8%	ns	2
	D 6%	ns	ns	3
	I 8%	ns	ns	5
	ns	n.d.	ns	6
	I 7%	n.d.	n.d.	8
	ns	ns	ns	7
	ns	ns	ns	9
Intensity >28,000	I 13%	I 10%	I 6%	1
	ns	D 19%	D 16%	10
	D 8%	D 9.5%	ns	4
	ns	D 10%	I 6%	2
	I 14%	ns	ns	3
	ns	ns	ns	5
	ns	n.d.	I 9%	6
	D 12%	n.d.	n.d.	8
	ns	I 9%	ns	7
	D 10%	ns	ns	9

The cells were treated with 1uM of KPT‐185, KPT‐330 or KPT‐8602 for 4 h and then harvested and examined as described (Materials and Methods). 3D nuclear telomere parameters were measured using TeloView (Vermolen et al., 2005). I, increase; D, decrease; ns, not significant; n.d., not done.

In conclusion, the 3D nuclear telomere architecture of tumor cells is preferentially disrupted by XPO1 inhibition, while that of normal cells was minimally or not affected suggesting a tumor cell–specific effect of XPO1 inhibition on nuclear architecture.

## Discussion

The nuclear architecture of cancer cell nuclei is unique. These nuclei undergo dynamic structural aberrations that include changes in their 3D organization of telomeres, chromosomes and chromosome orientation (Mai, 2010; Gadji et al., 2011; Righolt et al., 2011; Martin et al., 2013; Schmälter et al., 2015). This study demonstrates that the tumor cell‐specific 3D nuclear telomere organization is sensitive to XPO1 inhibition. Moreover, the effects of nuclear transport inhibition affect some tumor cells more than others. Lymphoid derived Jurkat cells are more sensitive to XPO1 inhibition by SINE than Raji cells while the breast cancer cells T47D are more resistant than HCC1937 cells.

Similarly, in our multiple myeloma patient study, each patient shows a distinct ex vivo response to a specific SINE compound. For example, KPT‐330 affects more 3D telomere parameters in patient 3 than in patient 2, while KPT‐8602 is better in patient 2 than in patient 3. Some patients respond well to more than one SINE compound (e.g., patient 1). These data reflect ex vivo responses of myeloma cells and would require patient studies to confirm the differential responses. If confirmed, these findings would suggest that a targeted choice of SINE compounds (KPT‐330, selinexor or KPT‐8602) could be made for specific patients and even enable successive use of one or the other if a broad response by more than one compound was seen. In addition the observed changes are rapid with the nuclear telomere organization changed significantly during a 4 h‐treatment window.

The current data suggest that the 3D nuclear organization of telomeres may be a sensitive monitor not only for SINE compounds downstream activity but for other novel drugs that are in development. If it is feasible to test patients' response ex vivo prior to the administration of a drug, appropriate personalized treatment management choices could be made and followed throughout the course of the treatment. For myeloma, this strategy would easily be achieved due to the ease of cell isolation and propagation in culture.

Cancer is a disease of DNA organization as already postulated more than a century ago by Boveri (1914, 1929). It is perhaps this aberrant order that is most vulnerable to XPO1 inhibition in tumor cells. Future investigations will have to determine why normal cells do not respond in the same way to XPO1. The tumor‐selective effect is an excellent opportunity for new ways to target cancer cells.
